# Supplementary material for: Highly parallelized droplet cultivation and prioritization of antibiotic producers from natural microbial communities
Source: eLife. 2021 Mar 25;10:e64774. doi: 10.7554/eLife.64774 (PMC8081529; doi:10.7554/eLife.64774)
Supplement: Supplementary file 1. [file elife-64774-supp1.docx]

Highly parallelized droplet cultivation and prioritization on antibiotic producers from natural microbial communities

Lisa Mahler^a,b^, Sarah P. Niehs^c^, Karin Martin^a^, Thomas Weber^a^, Kirstin Scherlach^c^, Christian Hertweck^c,b^, Martin Roth^a^, Miriam A. Rosenbaum^a,b^*

^a^Bio Pilot Plant, Leibniz Institute for Natural Product Research and Infection Biology - Hans Knöll Institute, 07745 Jena, Germany;

^b^Faculty of Biological Sciences, Friedrich Schiller University, 07743 Jena, Germany

^c^Biomolecular Chemistry, Leibniz Institute for Natural Product Research and Infection Biology - Hans Knöll Institute, 07745 Jena, Germany

*Correspondence: miriam.rosenbaum@leibniz-hki.de (M.A.R.)

SUPPLEMENTARY INFORMATION

**List of tables**

| 1 | Screenings for antibiotics in droplets with 2 different reporter strains and 2 different media… | 2 |
| --- | --- | --- |
| 2 | Diameter of inhibition zones for selected isolate D121-0906-b3-2-1………………………………………. | 2 |
| 3 | Media compositions for droplet cultivation of soil community……………………………………………….. | 2 |
| 4 | Media compositions for reporter strain cultivation…………………………………………………………………. | 3 |
| 5 | Media compositions for cultivation of isolates for antimicrobial activity testing……………………… | 4 |

|  | Medium | Reporter strain | Nb. of  isolates | Nb. of isolates characterized |
| --- | --- | --- | --- | --- |
| 1 | 50% CESE, 6% soy mannit medium | *E. coli* | 67 | 17 |
| 2 | 50% CESE, 6% malt medium | *E. coli* | 132 | 68 |
| 3 | 50% CESE, 6% soy mannit medium | *B. subtilis* | 180 | 63 |
| 4 | 50% CESE, 6% malt medium | *B. subtilis* | 78 | 9 |

**Table S 1** – Screenings for antibiotics in droplets with 2 different reporter strains and 2 different media.

**Table S 2** – Diameter of inhibition zones for selected isolate D121-0906-b3-2-1.

The antibiotics ^*1^Ciprofloxacin 5 g/mL and ^*2^Amphothericin B 10 g/mL were used as positive controls.

| Test strain | 0906-b3-2-1 | Control |
| --- | --- | --- |
| *Bacillus subtilis* | 15 | 28^*1^ |
| *Staphylococcus aureus* | 18/23 | 18^*1^ |
| *Escherichia coli* | 18 | 23/30^*1^ |
| *Pseudomonas aeruginosa* | 17 | 27/33^*1^ |
| *Mycobacterium vaccae* | 15 | 21^*1^ |
| *Sporobolomyces salmonicolor* | 13/20 | 18^*2^ |
| *Candida albicans* | 45/48 | 21^*2^ |
| *Penicillium notatum* | 45 | 19^*2^ |

**Table S 3** – Media compositions for droplet cultivation of soil community.

| Medium | Composition |
| --- | --- |
| SM | soy mannitol medium; 20 g/L soy coarse meal (Schkade Landhandel, Germany) + 20 g/L mannitol (Merck, Germany) in distilled water, pH adjusted to 6.5, 35 min at 121 °C |
| 0.06SM 0.5CESE | 6% (v/v) supernatant of soy mannitol medium (see above) + 50% (v/v) cold extracted soil extract + 44% (v/v) distilled water |

| Medium | Composition |
| --- | --- |
| TB + 1% glucose | 12 g/L tryptone (Bacto Tryptone, BD Bioscience, Belgium) + 24 g/L yeast extract (Bacto Yeast Extract, BD Bioscience, Belgium) + 4 g/L glycerol (Roth, Germany) in tap water, pH adjusted to 7.2 with NaOH, 20 min 121 °C, + 0.17 M KH_2_PO_4_ (Merck, Germany) + 0.72 M K_2_HPO_4_ (Merck, Germany) + 1% (w/v) glucose (VWR International, USA) |
| 2.5x TB + 1% glu-  cose | 30 g/L tryptone (Bacto Tryptone, BD Bioscience, Belgium) + 60 g/L yeast extract (Bacto Yeast Extract, BD Bioscience, Belgium) + 10 g/L glycerol (Roth, Germany) in tap water, pH adjusted to 7.2 with NaOH, 20 min 121 °C, + 0.425 M KH_2_PO_4_ (Merck, Germany) + 1.8 M K_2_HPO_4_ (Merck, Germany) + 1% (w/v) glucose (VWR International, USA) |

**Table S 4** – Media compositions for reporter strain cultivation.

| Medium | Composition |
| --- | --- |
| 0.3SM 0.2CESE | 30% (v/v) supernatant of soy mannitol medium (see above) + 20% (v/v) cold extracted soil extract + 50% (v/v) distilled water |
| 0.12SM 0.5CESE | 12% (v/v) supernatant of soy mannitol medium (see above) + 50% (v/v) cold extracted soil extract + 38% (v/v) distilled water |
| 0.5SM | 50% (v/v) supernatant of soy mannitol medium (see above) + 50% (v/v) distilled water |
| MMM | Modified Malt Medium; 2 g/L yeast extract (Bacto Yeast Extract, BD Bioscience, Belgium) + 2 g/L beef extract + 15 g/L malt extract in distilled water, pH adjusted with NaOH to 7.2, 20 min 121 °C |
| 0.5MMM 0.2CESE | 50% (v/v) modified malt medium (see above) + 20% (v/v) cold extracted soil extract + 30% (v/v) distilled water |
| NBE | 1 g/L beef extract + 2 g/L yeast extract (Bacto Yeast Extract, BD Bioscience, Belgium) + 5 g/L bact. peptone (Bacto Soytone, BD Bioscience, Belgium) + 5 g/L NaCl (Merck, Germany) in distilled water, 20 min 121 °C |
| NBE + 10Gluc | NBE (see above ) + 10 g/L glucose (VWR International, USA) |
| Soja2g | 15 g/L soy coarse meal (Schkade Landhandel, Germany) + 15 g/L glucose (VWR International, USA) + 5 g/L NaCl (Merck, Germany) + 1 g/L CaCO_3_ (Merck, Germany) + 0.3 g/L KH_2_PO_4_ (Merck, Germany) in distilled water, 20 min 121 °C |
| Soja2e | 20 g/L soy coarse meal (Schkade Landhandel, Germany) + 20 g/L glucose (VWR International, USA) + 5 g/L NaCl (Merck, Germany) + 1 g/L CaCO_3_ (Merck, Germany) in distilled water, 20 min 121 °C |
| M65 | 4 g/L yeast extract (Bacto Yeast Extract, BD Bioscience, Belgium) + 4 g/L glucose (VWR International, USA) + 10 g/L malt extract in distilled water, 20 min 121 °C |
| MGY M9 | 1 g/L (NH_4_)_2_SO_4_ (Merck, Germany) + 0.1 g/L MgSO_4_ (Roth, Germany) + 0.588 g/L Sodium Citrate (VWR International, USA) + 7 g/L KH_2_PO_4_ (Merck, Germany) + 2 g/L K_2_HPO_4_ (Merck, Germany) + 1.25 g/L yeast extract (Bacto Yeast Extract, BD Bioscience, Belgium) + 10 g/L glycerol (Roth, Germany) in distilled water, 20 min 121 °C |
| MGY + Gluc | MGY M9 (see above ) + 10 g/L glucose (VWR International, USA) |

**Table S 5** – Media compositions for cultivation of isolates for antimicrobial activity testing.
